# Associations between Housing Quality and Disability among Midlife and Older Adults

**DOI:** 10.1093/geroni/igaf122.393

**Published:** 2025-12-31

**Authors:** Kimberly Rollings, Philippa Clarke, Lu Qin, Sohel Ahmed, HwaJung Choi

**Affiliations:** University of Michigan, Ann Arbor, Michigan, United States; University of Michigan, Ann Arbor, Michigan, United States; University of Michigan, Ann Arbor, Michigan, United States; University of Michigan, Ann Arbor, Michigan, United States; University of Michigan, Ann Arbor, Michigan, United States

## Abstract

Numerous studies have linked housing quality to a variety of health outcomes throughout the life course. However, less is known about associations between housing quality and disability among midlife and older adults. This exploratory study examined these associations using biennial 2002 to 2018 data from the U.S. Health and Retirement Study (HRS). Available HRS housing data from a subsample of 28,357 community-dwelling adults aged 50 years and older (mean age=68.1 years, SD = 11.5 years) were harmonized and combined to create a housing quality scale (average of interviewer-reported and standardized: number of rooms per household member, and ratings of cleanliness, clutter, structural quality, maintenance, and safety features). Disability measures included any limitations in activities of daily living (ADL, e.g., walking, dressing, bathing) and instrumental activities of daily living (IADL, shopping, preparing meals), and any functional limitations (FL, e.g., difficulty climbing stairs). Logistic regression models indicated that a one unit increase in housing quality score (mean 0.03, range -2.56 to 2.05) was associated with up to 15% reduced odds of disability (any ADLs: OR = 0.88, 95% CI = 0.84-0.92; 2 any IADLs: OR = 0.87, 95% CI = 0.83-0.91; any FLs: OR = 0.85, 95% CI = 0.82 to 0.88) after accounting for housing type and ownership, and demographic, socioeconomic, health, and regional characteristics. These findings highlight a need for more rigorous research on housing quality and disability among midlife and older adults, and suggest that early and persistent interventions to address housing quality may support adults aging into and with disability and aging in place.

